# Variability in Distillers’ Co-Product Compositions and Their Nutritional Availability for Pigs: Insights from a Systematic Literature Review

**DOI:** 10.3390/ani14233455

**Published:** 2024-11-29

**Authors:** Herbert Rech, Alícia Zem Fraga, Carolina Haubert Franceschi, Alexandre Bonadiman Mariani, Caroline Romeiro de Oliveira, Gabriela Miotto Galli, Marie-Pierre Létourneau-Montminy, Luciano Hauschild, Teresinha Marisa Bertol, Ines Andretta

**Affiliations:** 1 Faculdade de Agronomia, Universidade Federal do Rio Grande do Sul, Porto Alegre 91540-000, RS, Brazil; herbertrech8@gmail.com (H.R.); aliciafraga@outlook.com.br (A.Z.F.); carolfranceschi3@hotmail.com (C.H.F.); alexandre.bonadiman.abm@gmail.com (A.B.M.); caroline_romeiro@outlook.com (C.R.d.O.); gabi-gmg@hotmail.com (G.M.G.); 2 Faculté des Sciences de l’Agriculture et de l’Alimentation, Université Laval, Québec, QC G1V 0A6, Canada; marie-pierre.letourneau-montminy.1@ulaval.ca; 3 School of Agricultural and Veterinarian Sciences, São Paulo State University, Jaboticabal 14884-900, SP, Brazil; luciano.hauschild@unesp.br; 4 Centro Nacional de Pesquisa de Suínos e Aves, Empresa Brasileira de Pesquisa Agropecuária—Embrapa, Concórdia 89715-899, SC, Brazil; teresinha.bertol@embrapa.br

**Keywords:** amino acids, DDGS, dried distillers grains with solubles, energy, feed ingredient, precision nutrition, swine

## Abstract

This study aimed to examine the nutritional diversity of distillers’ co-products in pigs. Two distinct databases were used in this study. The first was compiled through a systematic review of previous scientific publications that have evaluated the chemical composition and nutritional availability of these ingredients for pigs. The final database included information collected from 49 studies published between 2003 and 2022. Most of the studies focused on distillers’ co-products produced from corn (90%) and assessed dried distillers grains with solubles (DDGS; 92%). A wide variety of nutritional compositions was observed in the selected studies, with coefficients of variation of 8% for gross energy and 13% for crude protein in dried distillers grains (DDGs). In the general database, half of the studies were published from 2003 to 2015 and the other half from 2015 to 2022. Greater variability in the net energy content and standardized ileal digestible lysine concentrations from 2015 to 2022 were the main concerns. The second database included information on high-protein dried distillers grains (HP-DDGs) collected directly from several feed mills and was built using the results of chemical analyses. These results suggest that the nutritional matrix of co-products should be assessed for each processing plant. Nevertheless, a coefficient of variation of up to 6% was found for crude protein and up to 20% for ether extract. Variability in nutritional composition is an important challenge of using distillers’ co-products in pig feed.

## Simple Summary

This study aimed to evaluate the nutritional diversity of distillers’ co-products in pig diets. Two databases were used. The first, based on 49 scientific studies from 2003 to 2022, focused mainly on corn-derived distillers’ co-products, with most analyzing dried distillers grains with solubles (DDGS). Variations in the nutritional composition were noted, especially in the net energy and digestible lysine content, from 2015 to 2022. The second database, created from field surveys in Brazil (n = 1550), analyzed the DDGS samples. The results highlight significant variability in the crude protein and ether extract, emphasizing the need for plant-specific nutritional assessments to improve the sustainability of pig production.

## 1. Introduction

Consistent information on animal nutrient requirements and feedstuff characteristics is non-negotiable when proposing precise feeding programs [1]. This information allows nutritionists to determine a given amount of feedstuffs that will provide a diet that will allow production goals to be achieved at an optimized feed cost. Feed formulations can be established using published values of ingredient composition, such as the NRC [2] and Rostagno et al. [3]. However, using table values or other static compositions is a risky practice that can lead to inaccurate formulations owing to the wide variability in the nutritional composition and digestibility of feed ingredients.

Dried distillers grains (DDGs) are major co-products of ethanol production and have been widely used in the animal feed industry because of their high nutritional content and low production cost [4]. The various raw materials used in alcoholic fermentation include corn, wheat, barley, sorghum, triticale, and even mixtures of these ingredients. In addition, the origin of the raw materials [5] and the type or duration of the fermentation process [6] can potentially contribute to significant variations in their nutritional composition and digestibility.

The potential use of distillers’ co-products in pig diets has been highlighted as an important source of protein and energy [7,8]. A recent literature review found that most studies reported no changes in performance when distillers’ co-products were used to partially replace corn, soybean meal, and inorganic phosphorus [8]. However, the variability in raw materials and the discrepancies in their nutritional composition may affect the accuracy of feed formulations and animal growth performance if not considered by animal nutritionists.

Understanding the variability in the nutritional composition of distillers’ co-products is essential for implementing precise feeding programs. Precisely formulated feeds can improve nutrient-use efficiency, a key step toward enhancing the sustainability of animal production. Therefore, the present study was designed to evaluate the variability in the chemical characteristics of several types of distillers’ co-products and their relationship with energy and amino acid (AA) availability in pigs.

## 2. Material and Methods

The investigations were conducted through a systematic literature review (database I), and, given the fast-growing importance of distillers’ co-products in Brazil, a dataset from the main manufacturers in the Brazilian market was also assessed (database II).

### 2.1. Database I: Information from the Literature

The digital databases PubMed, Web of Science, and Scopus were searched in January 2023 to identify studies reporting trials in which pigs were fed distillers’ co-products. The review question was proposed using the “PICo” framework, which indicates “population”, “interest”, and “context”. Thus, a set of keywords was combined to include elements designating the population (e.g., pigs), interest (e.g., distillers grains), and context (e.g., nutritional composition) of this research. A final search was performed using the terms: (pig OR pigs OR swine OR piglet*) AND (“dried distillers*” OR “DDGS” OR “DDG” OR “dried distillers grains with soluble” OR “distiller grain*” OR “distillers”) AND (digestibility OR digestible* OR “apparent ileal digestibility” OR “standardized ileal digestibility” OR performance).

**Figure 1:**
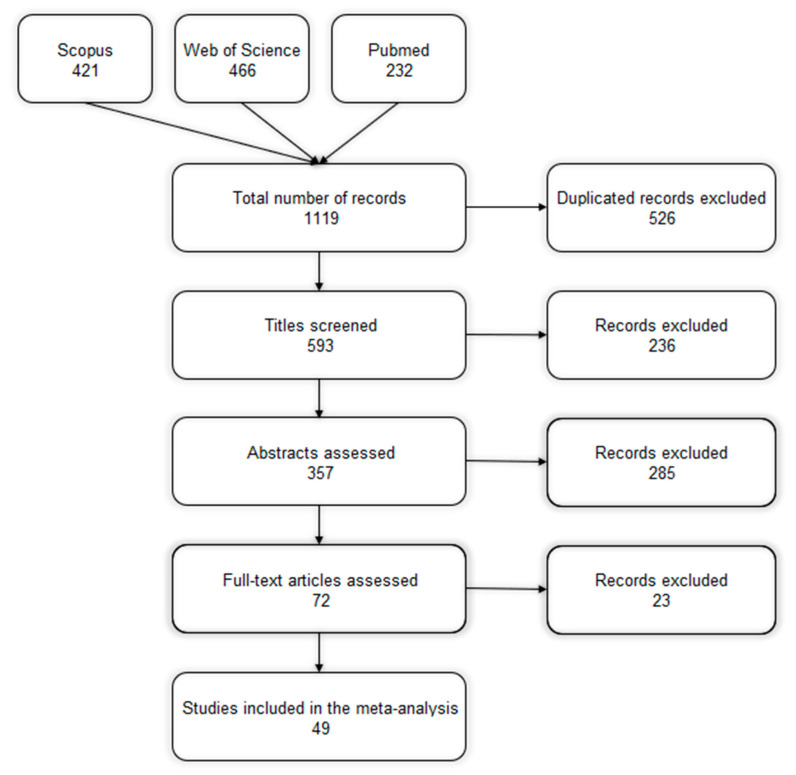
Prisma flow diagram describing the study selection process for the database.

All references obtained in each database were exported to EndNote X9 (Clarivate Analytics, CA), where duplicate studies (from distinct databases) were eliminated based on the title and year of publication. The title and abstract of each result were independently reviewed by two researchers to select papers that would be fully evaluated. The full versions of the selected studies were critically evaluated by the same researchers in terms of their quality and relevance, considering the systematic review’s objectives. The criteria used in these steps were as follows: (i) studies published in indexed journals, considering their acceptance for publication as a subjective criterion for their methodological quality; (ii) in vivo assessment of distillers’ co-products for pigs; (iii) characterization of the raw material, including the dry matter (DM) content or relative values; (iv) digestibility of AA and/or energy; and (v) publications from 2003. Any removal was registered in a PRISMA flow diagram (Figure 1).

Data were obtained from the studies and organized in a digital file. Each row of the spreadsheet represented a treatment from the original publication, which, in most cases, was a sample of the tested ingredient. Each column represented a variable such as general information (e.g., the author’s last name, year of publication, and country), the experimental design (e.g., replacing the basic diet, indigestible markers, initial body weight, sexual category, and number of replicates), the type of distillers’ co-product, the raw material used for fermentation, and characterization of the ingredients (energy, total AA, apparent ileal digestible (AID) AA, and standardized ileal digestible (SID) AA content). All chemical composition data were converted and standardized on a DM basis.

The dataset was categorized into 6 group-based products: DDGs—dried distillers grains; DDGS—dried distillers grains with solubles; HP-DDGS—high-protein dried distillers grains with solubles; HP-DDGs—high-protein dried distillers grains; FWS—fiber with solubles; HYP—high yeast and protein. An additional classification into 5 groups was performed based on the grain used: corn, wheat, sorghum, triticale, and mixtures (e.g., rice, corn, wheat, and sorghum blends).

### 2.2. Database II: Information from the Brazilian Field Survey

An extensive field survey was performed considering the main producers in Brazil, which is an emerging market for distillers’ co-products. In order to establish more accurate characterizations, 1550 samples of DDGS and HP-DDGs were collected directly from several feed mills, and the results of the chemical analysis were used to build the database.

Sampling was performed from October 2017 to December 2022 in major feed industries located in the central-west and southeast regions of Brazil, in which nearly all pig production in Brazil occurs. The exact location of the companies is not presented to preserve the identity of the companies (supplier and final user). Random samples were collected directly from trucks (at different points and depths using conventional samplers) as the material entered the feed mills. Samples from the same truck were homogenized and combined into a representative sample of the entire load (500 g). Each sample was identified according to the factory and manufacturing unit in which it was produced.

Crude protein (CP), ether extract (EE), ash, crude fiber (CF), acid detergent fiber (ADF), and neutral detergent fiber (NDF) analyses were performed by near-infrared spectroscopy (NIRS) analysis, and protein solubility (PS) analyses determined the soluble protein in KOH 0.036M solution, which was later quantified by the Kjeldahl method [9]. All results were expressed on a DM basis for standardization.

Data from four companies and six factories were obtained. The massive availability of co-products from Brazilian distilleries is a recent occurrence, and therefore, a large part of the database was composed of samples from two suppliers. A subset was created using these data to further explore the variability among factories of the same company, considering samples of batches from 2018 to 2022 of only HP-DDGs produced from corn (which comprised the vast majority of the database). The factories are reported hereafter by sequential numbers separated by a dot (i.e., Factory 1.1; Factory 1.2; Factory 2.1), where the first number of the origin code corresponds to the company and the second to factory. Despite being part of the same company, these factories were located far from each other (in some cases, in different states) and, for this reason, the comparison was worth investigating.

### 2.3. Statistical Analysis

Descriptive statistics (the frequency, mean, minimum, maximum, and coefficient of variation—CV) and graphical analysis were used to characterize the databases. The Levene and Cramer–von Mises tests were used to verify the homogeneity of the variances and normality of the studentized residuals, respectively. The sample (the data of a single distiller’s co-product load assessed in the original study) was considered the experimental unit. For multiple comparison analysis (e.g., HP-DDGs among factories and manufacturing units), the means of groups with homogeneous variance were compared using ANOVA followed by the Tukey–Kramer test, whereas the means of groups with heterogeneous variance were compared using a non-parametric test (Kruskal–Wallis test), followed by multiple comparisons of the mean rank for all groups according to Siegel and Castellan (1988). In both cases, the model was *Yik = µ + MAi + eik*, where *Yik* is the observed variable (CP, EE, Ash, NDF, or ADF), *µ* is the mean, *MAi* represents the effect of the manufacturers (Factory 1.1, Factory 1.2, and Factory 2.1), and *eik* is considered the error. All statistical analyses were performed using Minitab (v. 21, Minitab Inc., State College, PA, USA) and Statistica (v. 10, StatSoft Inc., Minneapolis, MN, USA) software. The results were interpreted as statistically significant if the *p*-value is <0.05.

## 3. Results and Discussion

### 3.1. Systematic Review

A total of 1119 references were identified in the online literature search, of which 526 duplicates were removed with the assistance of the reference manager. Other references were removed during the title (n = 236) and abstract (n = 285) evaluation. The main criterion used in these two steps was to maintain studies on dietary supplementation with distillers’ co-products for pigs. In addition, 23 references were excluded after the full evaluation due to specific criteria related to the methodological aspects of the original studies (e.g., failure to present the DM content of the ingredient or missing information on the raw material used for distillers’ co-product production). Finally, 49 studies matched all the criteria and were included in the database (Figure 1).

Most samples considered in the database (Table 1) were collected in the USA (62%), followed by China (24%) and Canada (6%). The studies included 891 and 883 replicates for AA and energy, respectively. The animal body weight (BW) was lower than 30 kg in only 12% of the studies and was higher than 60 kg in 22% of the studies.

Most distillers’ co-product types included in the current systematic literature review were DDGS (92% of the total database). Indeed, DDGS is the main co-product of cereal-based ethanol production [10]. The greater use of DDGS in pigs diets in recent years has been associated with increased ethanol production (as a partial substitute for petroleum) [4], and, in some price contexts, its cost competitiveness compared to corn and soybean meal [11]. Notably, the majority of studies reviewed lacked detailed descriptions of the production processes. This limitation is significant in this field, as production methods heavily influence the characteristics of the resulting products. Understanding these processes in detail would enable a more thorough analysis of how different factors impact nutrient availability in by-products.

Regardless of the type of distillers’ co-product, corn was the main raw material in the studies. Although corn is the predominant source of fermentable starch with the highest ethanol yield [12], this result may be associated with the number of samples from the USA presented in the database, where distillers’ co-product production is primarily based on corn [10]. For instance, only 25% of the European samples assessed were produced from corn.

**Figure 2:**
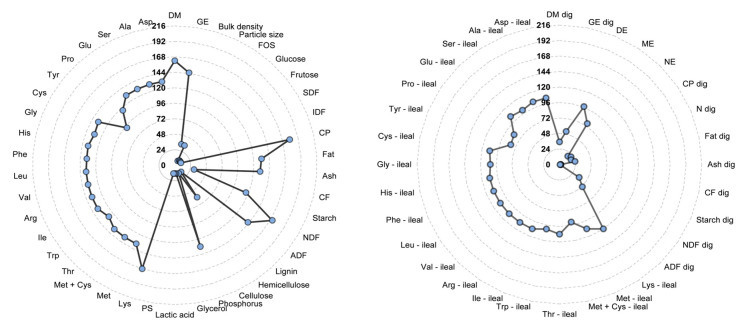
Frequency of analysis of studied samples. DM = dry matter; GE = gross energy; DE = digestible energy; ME = metabolizable energy; NE = net energy; FOS = fructooligosaccharides; SDF = soluble diet fiber; IDF = insoluble diet fiber; CP = crude protein; Fat = ether extract; CF = crude fiber; NDF = neutral detergent fiber; ADF = acid detergent fiber; PS = protein solubility in KOH; Lys = lysine; Met = methionine; Thr = threonine; Trp = tryptophan; Ile = isoleucine; Arg = arginine; Val = valine; Leu = leucine; Phe = phenylalanine; His = histidine; Gly = glycine; Cys = cystine; Tyr = tyrosine; Pro = proline; Glu = glutamine; Ser = serine; Ala = alanine; Asp = asparagine. Dig: digestible analyses. Amino acid with the abbreviation “ileal” is standardized ileal digestible (SID) AA; AA without abbreviations refers to total content.

Information on analyzed CP and NDF were present in 83 and 79% of the total samples studied (n = 216), respectively (Figure 2). Furthermore, 67% of the samples were analyzed for gross energy (GE), 45% for digestible energy (DE), 35% for metabolizable energy (ME), and 8% for net energy (NE). The gross energy content of a feed ingredient is relatively easy to measure, which may explain the greater number of samples analyzed for this variable. Energy is the most expensive component of a pig diet. Therefore, accurate estimates of the energy content of feed ingredients are important [13]. Although the NE system more accurately represents the energy available to animals compared to the DE or ME [14], the NE content of ingredients is influenced by several factors, mainly by the estimation method (comparative slaughter, indirect calorimetry technique, or equations; [15]. However, because of differences in the experimental design (environmental conditions, housing, density, etc.), characteristics of the animals (age, stage of growth, etc.), and variability in the chemical composition of the ingredients, there are differences in the estimates of the NE values between the methods [15]. Most NE values presented in the studies were obtained from equations, which led to high correlations between the GE and NE (r = 0.748), the DE and NE (r = 0.908), and the ME and NE values (r = 0.859). In terms of comparison, the correlations between the GE and DE (r = 0.345) and between the GE and ME (r = 0.296) were remarkably lower.

In the current systematic review, 78% of the studied samples had analyzed information on the total Lys, whereas 55% had analyzed information on the SID Lys. Indeed, the total AA concentrations were more frequent than the SID values for all studied AAs. It is well known that the total AA content of a given feed ingredient may not be completely absorbed, and not all absorbed AA are metabolically available [2]. Basal endogenous losses of AA include sloughed intestinal epithelial cells, mucin protein, and digestive enzyme secretions [16]. As SID coefficients of AAs are calculated by subtracting basal endogenous AA losses from the ileal outflow of AA [17], SID values in a diet formulation are accepted as the preferred measure of digestible AA [18]. However, this is an expensive and time-consuming method, with a series of ethical concerns.

New technologies in ethanol-processing plants allow for the development of different types of co-products. The wide range of co-products available for use in animal feed requires an even more accurate evaluation of their nutritional value [19]. To explore the variability among samples from the literature review according to the type of co-product and source (grain), the nutritional compositions of distillers’ co-products were assessed and are presented in Table 2 (complementary information can be found in Tables S1 and S2). Irrespective of the source and, as expected, a greater average value of CP was observed in HP-DDGs than in DDGS (average difference of 131.7 g/kg). During the production processes, a fractionation step of grains may be added before fermentation to remove germ and fiber (nonfermentable fractions; Mohammadi et al., 2021). As a result, the protein content is concentrated in the final product, which is marketed as HP-DDGs [12]. Regarding the fiber content, the mean NDF and ADF of DDGS were 368.4 and 129.5 g/kg, respectively (Table 2). These values are comparable with those previously reported [19], which were 377.6 g/kg (NDF) and 159.9 g/kg (ADF). Greater coefficients of variation (CVs) were found for both NDF (25% of HP-DDGs) and ADF (35% of DDGS). It should be noted that most of the fiber present in DDGS is insoluble, for which the main fermentation site is the colon [20]. Therefore, although some studies have reported improvement in the intestinal health of pigs, with the inclusion of DDGS [21,22], greater insoluble fiber levels in the form of DDGS were also associated with the decreased digestibility of dietary components, such as DM, EE, and AA [23].

As mentioned above, distillers’ co-products from corn had the greatest frequency in the studies (90% of the studied samples that comprised the current database). Other ingredients have a seasonal and lower-volume supply, which would generate instability in ethanol production. This may explain the combined frequency of 1.4% of the studies on the distillers’ co-products of sorghum and triticale. Obviously, variability related to the type of grain is expected, as they differ in their composition before processing. Unfortunately, the small number of samples from sources of DDGS other than corn-based precludes any comparison in terms of their nutritional composition and variation.

Distillers’ co-products from corn had the greatest frequency in the studies (90% of the studied samples that comprised the current database). Sorghum and triticale have a seasonal and low-volume supply, which would generate instability in ethanol production. This may explain the combined frequency of 1.4% of the studies on the distillers’ co-products of sorghum and triticale. Obviously, we can expect variability related to the type of grain, as they differed in their compositions before processing. Unfortunately, the small number of samples from sources of DDGS other than corn precludes any comparison in terms of their nutritional composition and variation.

As expected, CP showed great variability between the DDGS and HP-DDGs (Table 3), being directly affected by differences in the technological processes of fermentation [24]. On the other hand, the mean EE contents in the DDGS and HP-DDGs were relatively uniform because of industrial extraction controls. Even so, high variability coefficients were found for EE in the DDGS (36%) and HP-DDG samples (46%). Surprisingly, the variability coefficients found for GE were remarkably lower than those for EE (8.2 and 15.4 times lower for DDGS and HP-DDGs, respectively). The variability in the DE and ME values observed in the HP-DDGS samples was significantly higher than that in the DDGS (3.4 and 3.1 times greater for DE and ME, respectively). This condition highlights the importance of having tools to consider the variability not only in the gross composition but also in more intrinsic traits, such as the availability of nutrients to the animals.

**Figure 3:**
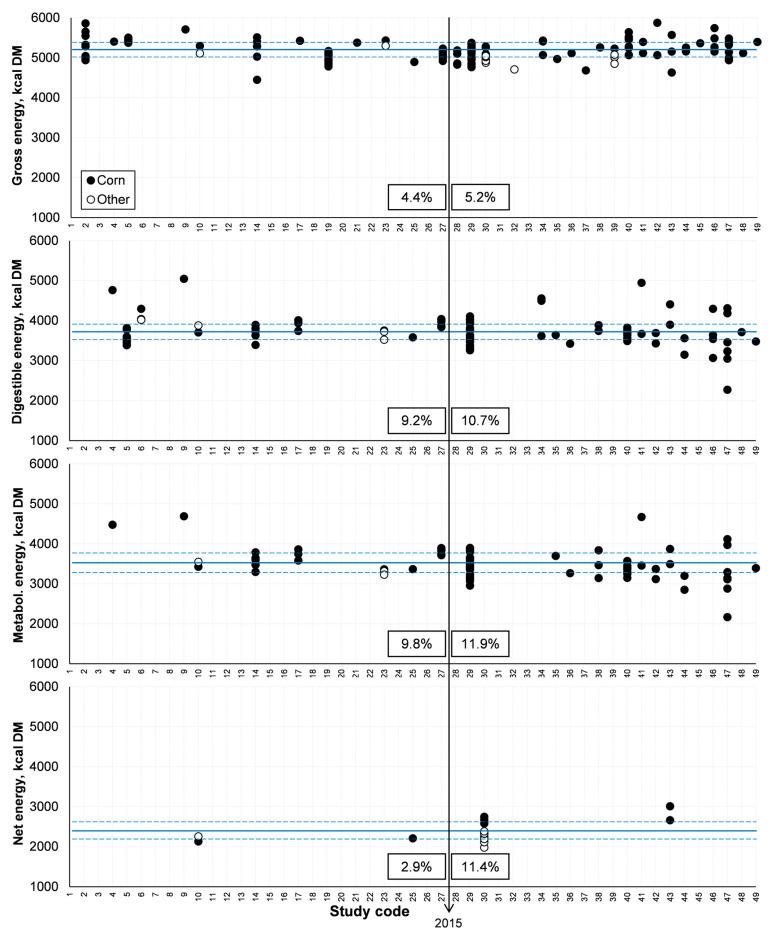
Variability in energy content among studies. Energy expressed in kcal/kg of DM. GE = gross energy; DE = digestible energy; ME = metabolizable energy; NE = net energy. The year 2015 is highlighted because half of the studies that comprised the current systematic literature review were published from 2003 to 2015 and the other half from 2015 to 2022. The numbers indicated correspond to the CV (%) up to and since 2015, respectively. Lines indicated the average composition, and dotted lines delimited the interquartile range.

**Figure 4:**
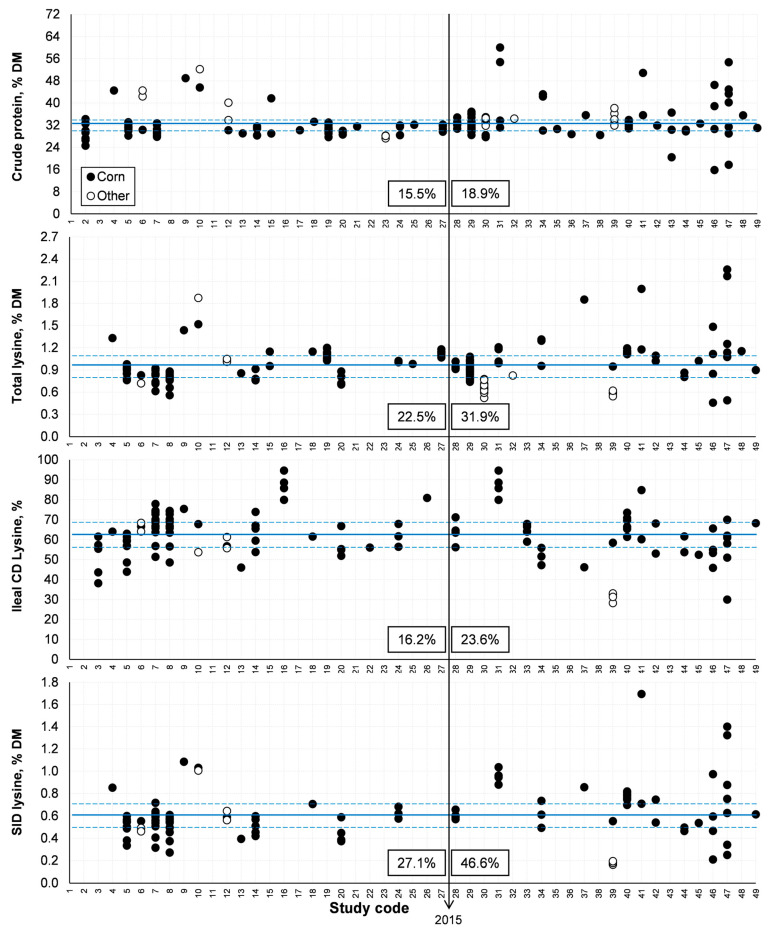
Variability in crude protein and lysine contents among studies. CD = coefficient of digestibility; SID = standardized ileal digestibility. The year 2015 is highlighted because half of the studies that comprised the current systematic literature review were published from 2003 to 2015 and the other half from 2015 to 2022. The numbers indicated correspond to the CV (%) up to and since 2015, respectively. Lines indicated the average composition, and dotted lines delimited the interquartile range.

From the general database, half of the studies were published from 2003 to 2015 and the other half from 2015 to 2022 (Figure 3 and Figure 4, respectively). The CV values were lower from 2003 to 2015 than from 2015 to 2022 for all studied variables. The greater variability after 2015 may be explained by the increasing implementation of industries and the consequent diversification of fermentation processes to produce ethanol. Thermal processing during drying is one of the steps in co-product production that deserves special attention. If excess heat is applied, Lys may be damaged, which in turn precludes its use in protein synthesis [12,13,14,15,16,17,18,19,20,21,22,23,24,25]. Eventual damage to some samples in the studies that comprised the overall database could explain the greater variation in SID Lys (a CV of 47% after 2015) compared to total Lys (a CV of 32% in the same period). This is a relevant approach because the SID value of Lys is representative of distillers’ co-products’ nutritional quality [12].

### 3.2. Brazilian Database

Brazil is the second-largest producer of ethanol but has historically used sugarcane as the primary feedstock [26]. However, in recent years, ethanol has also been produced from corn, mainly in regions with large production areas. To exclude the effects of different sources, the subsequent analysis was based only on the chemical analysis of corn DDGS and HP-DDG samples produced in Brazil and identified during a field survey (Brazilian database; Table 4). Regarding DDGS, differences in the nutritional composition were observed between the Brazilian samples and the data previously published by [27]. Although both studies used corn as the raw material, the differences in the EE and NDF between the studies were 103 and 175 g/kg, respectively. However, the results for the Brazilian HP-DDGs obtained in this study are comparable with those described by [26], whose average values of CP, EE, Ash, NDF, and ADF for HP-DDGs from Brazil were 433, 119, 260, 307, and 107 g/kg, respectively.

The nutritional values for DDGS and other co-products, such as HP-DDGs, were not included in the ingredient list considering the previous version of the Brazilian Tables for Swine and Poultry, but DDGS and HP-DDGs were considered in the most recent version of the reference tables [3]. These recent results are comparable to those presented in the current survey, despite the great difference in the number of samples considered (e.g., the CP results presented in the Brazilian Tables were obtained from 13 samples of DDGS and 14 samples of HP-DDGs).

The data from the main Brazilian manufacturers (a subset of the Brazilian database) were further studied to identify possible differences in the nutritional composition of HP-DDGs among factories (namely Factory 1 and Factory 2) and/or manufacturing units (units) of the same factory. There were differences among the factories and units for all the studied variables (*p* < 0.001; Table 5). These findings indicate that the characterization of distillers’ co-products should be evaluated for each manufacturing unit. Indeed, each ethanol production unit has specific technologies in its fabrication processes (such as the fermentation, distillation, and/or drying methods) [28] at different industrial scales. Therefore, the combination of different technologies and processes to optimize ethanol production in each manufacturing unit may be another reason explaining the variation in the nutrition profiles.

**Figure 5:**
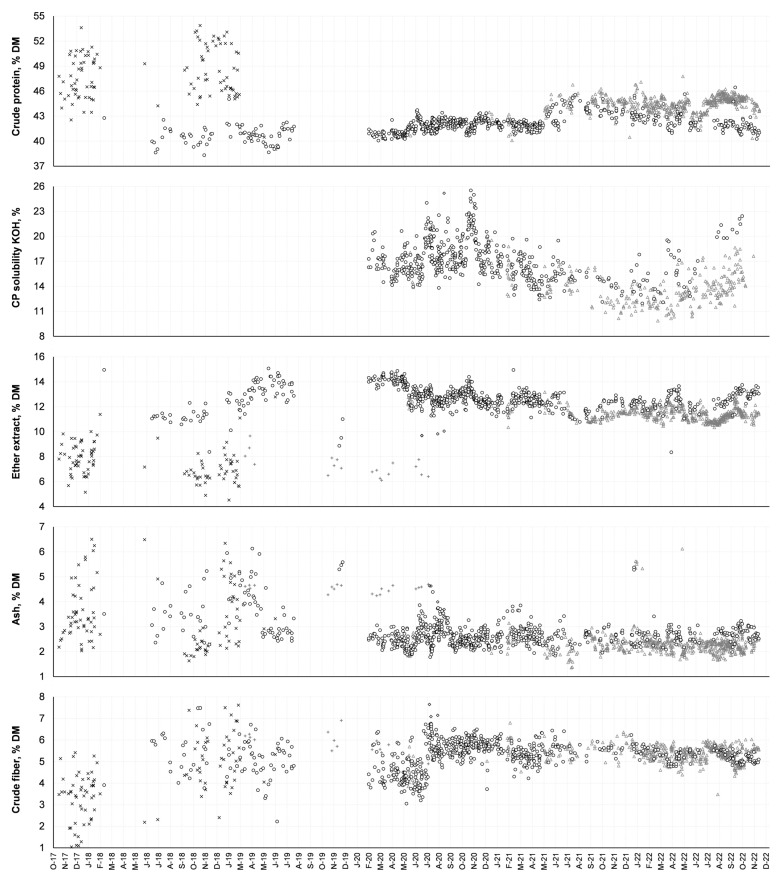
Variation in the nutritional compositions of HP-DDG corn samples collected at different time points from Brazilian suppliers. CP = crude protein; DM = dry matter; PS = protein solubility in KOH. The letters represent the months, and the last two numbers refer to the year. Different symbols indicate different suppliers.

Samples of corn HP-DDGs from the four Brazilian suppliers were collected at different time points. The KOH test indirectly determines the degree of protein denaturation due to heat processing [29]. Thus, this test is used to evaluate the quality of the thermal and mechanical processing of a feedstock (e.g., the KOH solubility generally decreases as the heat treatment increases) [30]. Whereas the variability in the CP (Figure 5; in % of DM) was reduced over time, the variability in the CP’s solubility in KOH was maintained. However, many factors inherent to the assay can affect protein solubility, including particle size, extraction duration, agitation velocity, and rotation time [31]. The data show an increase in CP after 21 May in a manufacturing unit, which was not followed by other units of other companies. This variation is possibly related to process optimization that, at the exact moment of these variations, could cause difficulties in optimizing the formulation if there is no intensive analytical monitoring in the feed mills.

Managing the variability in the nutritional compositions of feed ingredients is a challenge for animal nutritionists. Proper evaluation and adjustment are crucial to avoid waste, which has significant environmental and economic implications. By understanding and mitigating this variability, nutritionists can ensure more efficient and sustainable pig production. By exploring the variability in the data obtained from the literature and the field, this study emphasizes the importance of precise nutritional assessments, which can lead to improved feed formulations, reduced environmental impact, and enhanced economic viability within the pig production chain.

Future research should focus on better understanding the factors that influence the variability in nutritional compositions among processing plants, especially in terms of the net energy and digestible amino acids. In addition, standardized methods for assessing nutritional compositions should be developed to reduce inconsistencies. Finally, research could focus on gut and blood microbiota-associated health, which is another gap in the literature.

## 4. Conclusions

Notable variation in the chemical compositions of the samples was observed across both databases (the literature dataset and the field survey). Variability among primary types and suppliers underscores the critical need for standardized testing protocols and regular monitoring of co-product composition. Such practices would support the precise formulation of animal feeds, ensuring that nutritional requirements are met. These findings also highlight the importance of ongoing research and development in feed ingredient evaluation and quality control.

## Figures and Tables

**Table 1:** Description of studies focusing on the nutritional value of distillers’ co-products for pigs.

Code ^1^	Author	Year	Country ^2^	Digestibility ^3^	Type ^4^	Source ^5^
1	Liang	2003	China	Ileal	DDGs	Corn
2	Guo	2004	China	Ileal	DDGS	Corn
			China	Total	DDGS	Corn
			China	Total	DDGs	Corn
			China	Total	DDGS	Corn
3	Fastinger and Mahan	2006	USA	Ileal	DDGS	Corn
4	Widmer	2007	USA	Ileal and total	HP-DDGs	Corn
5	Stein	2006	USA	Ileal and total	DDGS	Corn
6	Widyaratne and Zijlstra	2007	Canada	Ileal and total	DDGS	Mix
			Canada	Ileal and total	DDGS	Wheat
			USA	Ileal and total	DDGS	Corn
7	Pahm	2008	USA	Ileal	DDGs	Corn
			USA	Ileal	DDGS	Corn
8	Pahm	2009	USA	Ileal	DDGS	Corn
9	Kim	2009	USA	Ileal and total	HP-DDGs	Corn
10	Jacela	2010	USA	Ileal and total	HP-DDGs	Corn
			USA	Ileal and total	HP-DDGS	Sorghum
11	Urriola and Stein	2010	USA	Ileal and total	DDGS	Corn
12	Yang	2010	Canada	Ileal	DDGS	Corn
			Canada	Ileal	DDGS	Mix
			Canada	Ileal	DDGS	Wheat
13	Almeida	2011	USA	Ileal	DDGS	Corn
14	Ren	2011	China	Ileal and total	DDGS	Corn
15	Almeida and Stein	2012	USA	Total	DDGS	Corn
16	Adeola and Ragland	2012	USA	Total	HP-DDGs	Corn
			USA	Ileal	DDGs	Corn
			USA	Ileal	DDGS	Corn
			USA	Ileal	HP-DDGs	Corn
			USA	Ileal	HP-DDGS	Corn
17	Liu	2012	USA	Total	DDGS	Corn
18	Soares	2012	USA	Ileal	DDGS	Corn
19	Kerr	2013	USA	Total	DDGS	Corn
20	Almeida	2013	USA	Ileal	DDGS	Corn
21	Baker	2013	USA	Total	DDGS	Corn
22	Petersen	2014	USA	Ileal	HP-DDGs	Corn
23	Adeola and Kong	2014	USA	Total	DDGS	Corn
24	Curry	2014	USA	Total	DDGS	Sorghum
			USA	Total	DDGS	Triticale
			USA	Total	DDGS	Corn
25	Graham	2014	USA	Ileal and total	DDGS	Corn
26	Adebiyi	2015	USA	Ileal	DDGS	Corn
27	Kerr	2015	USA	Total	DDGS	Corn
28	Li	2015a	China	Ileal	DDGS	Corn
29	Li	2015b	China	Total	DDGS	Corn
30	Tanghe	2015	Austria	Ileal and total	DDGS	Mix
			Belgium	Ileal and total	DDGS	Mix
			France	Ileal and total	DDGS	Wheat
			Germany	Ileal and total	DDGS	Mix
			Hungary	Ileal and total	DDGS	Corn
			Netherlands	Ileal and total	DDGS	Corn
			Netherlands	Ileal and total	DDGS	Wheat
			Spain	Ileal and total	DDGS	Corn
31	Adeola and Ragland	2016b	USA	Ileal	DDGs	Corn
			USA	Ileal	DDGS	Corn
			USA	Ileal	HP-DDGs	Corn
			USA	Ileal	HP-DDGS	Corn
32	Agyekum	2016	Canada	Ileal and total	DDGS	Mix
33	Kim	2017	USA	Ileal	DDGS	Corn
34	Rho	2017	Canada	Ileal	DDGS	Corn
			Canada	Ileal	HP-DDGS	Corn
			Canada	Ileal	HP-DDGS	Corn
35	Huang	2018	USA	Total	DDGS	Corn
36	Navarro	2018	USA	Total	DDGS	Corn
37	Park	2018	USA	Ileal	DDGS	Corn
38	Xie	2019	China	Total	DDGS	Corn
39	Curry	2019	USA	Ileal	DDGS	Corn
			USA	Ileal	DDGS	Mix
			USA	Ileal	DDGS	Wheat
40	Espinosa	2019	USA	Ileal and total	DDGS	Corn
41	Cristobal	2020	USA	Ileal and total	DDGS	Corn
			USA	Ileal and total	HP-DDGS	Corn
42	Rodriguez	2020	USA	Ileal and total	DDGS	Corn
43	Boucher	2021	Canada	Total	DDGS	Corn
			Canada	Total	FWS	Corn
			Canada	Total	HP-DDGs	Corn
44	Acosta	2021	USA	Ileal and total	DDGS	Corn
45	Park	2021	USA	Ileal	DDGS	Corn
46	Paula	2021	Brazil	Ileal and total	DDGS	Corn
			Brazil	Ileal and total	FWS	Corn
			Brazil	Ileal and total	HP-DDGs	Corn
47	Yang	2021	USA	Ileal and total	DDGS	Corn
			USA	Ileal and total	FWS	Corn
			USA	Ileal and total	HP-DDGs	Corn
			USA	Ileal and total	HYP	Corn
48	Zangaro and Woyengo	2022	USA	Ileal and total	DDGS	Corn
49	Zhang	2022	USA	Ileal and total	DDGS	Corn

^1^ Codes were attributed in a chronological sequence to represent each study considered in the current systematic review and are the same as those presented in the next tables and figures. ^2^ Considering the countries where the samples were collected. ^3^ The main criteria used for sample collection, if presented. ^4^ The types of distillers’ co-products considered in the current paper. DDGs: dried distillers grains; DDGS: dried distillers grains with solubles; FWS: fiber with solubles; HP-DDGs: high-protein dried distillers grains; HP-DDGS: high-protein dried distillers grains with solubles; and HYP: high yeast and protein. ^5^ Source: raw materials used to produce the distillers’ co-products.

**Table 2:** Characterization of distillers’ co-products by type and grain source used for fermentation ^1^.

	n ^2^	%	GE ^1^	CP	EE	Ash	NDF	ADF	Starch
**Type ^3^**									
DDGs	10	4.6	5384 (8.1)	307.1 (13.1)	95.1 (*)	34.5 (60.1)	494.6 (17.0)	266.2 (35.2)	43.52 (*)
DDGS	183	84.7	5147 (4.4)	314.5 (6.5)	90.0 (36.0)	54.6 (20.9)	368.4 (15.8)	129.5 (34.9)	81.95 (65.2)
FWS	3	1.4	4925 (5.4)	180.0 (12.8)	101.6 (28.9)	52.7 (5.5)	385.8 (13.5)	108.8 (35.0)	77.92 (*)
HP-DDGs	12	5.6	5488 (3.0)	446.2 (14.7)	69.2 (46.1)	27.6 (27.3)	406.0 (24.7)	210.0 (28.2)	71.9 (90.5)
HP-DDGS	6	2.8	5333 (0.3)	486.6 (12.6)	55.6 (76.1)	43.3 (69.6)	316.2 (1.4)	167.7 (7.5)	13.21 (13.5)
HYP	2	0.9	5414 (1.7)	497.9 (14.0)	99.8 (70.0)	55.3 (11.1)	184.3 (62.0)	89.0 (58.0)	-
**Source ^4^**									
Corn	194	89.8	5199 (4.7)	322.4 (17.7)	89.7 (38.2)	51.9 (27.0)	372.8 (19.0)	142.5 (42.4)	80.0 (66.6)
Mix	12	5.6	4971 (2.4)	344.5 (7.8)	81.2 (28.6)	57.4 (13.0)	322.9 (9.6)	172.3 (24.3)	20.6 (37.7)
Sorghum	2	0.9	5201 (2.5)	402.0 (42.4)	61.5 (63.1)	54.3 (*)	304.2 (38.6)	195.7 (4.4)	-
Triticale	1	0.5	5298 (*)	272.6 (*)	56.4 (*)	-	357.2 (*)	153.3 (*)	-
Wheat	7	3.2	4976 (1.3)	367.1 (12.4)	64.4 (37.3)	49.7 (13.3)	321.2 (16.5)	198.9 (37.7)	64.9 (85.8)

GE = gross energy; CP = crude protein; EE = ether extract; NDF = neutral detergent fiber; ADF= acid detergent fiber; ^1^ Except for GE, whose unit of measurement is kcal/kg, the other analyses are expressed in g/kg of DM. The CV (%) is shown in parentheses. Variables with only one sample are indicated with an asterisk (*). ^2^ n = number of samples. ^3^ Types of distillers’ co-products considered in the current paper. DDGs: dried distillers grains; DDGS: dried distillers grains with solubles; FWS: fiber with solubles; HP-DDGs: high-protein dried distillers grains; HP-DDGS: high-protein dried distillers grains with solubles; and HYP: high yeast and protein; ^4^ Source: raw materials used to produce the distillers’ co-products.

**Table 3:** Variability of corn DDGS and HP-DDGs among countries ^1^.

Type ^2^		DDGS								HP-DDGs			
Country		USA	China	Netherlands	Hungary	Spain	Canada	Brazil	All Groups	USA	Canada	Brazil	All Groups
**GE**	Mean	5198	5120	5255	5278	5255	5108	5265	5168	5423	5568	5611	5488
	n ^3^	59	42	1	1	1	2	1	107	5	1	2	8
	CV ^4^	4.5	4.3	*	*	*	1.2	*	4.4	3	*	3.2	3
**DE**	Mean	3650	3677	-	-	-	3755	3538	3663	3846	4405	3966	3946
	n	41	33	-	-	-	2	1	77	5	1	2	8
	CV	6.8	6.4	-	-	-	5.3	*	6.5	28.8	*	11,7	22.3
**ME**	Mean	3444	3489	-	-	-	3494	-	3468	3611	3872	-	3654
	n	29	33	-	-	-	1	-	63	5	1	-	6
	CV	8.3	7.9	-	-	-	-	-	8	28.1	*	-	25
**NE**	Mean	2208	-	2747	2675	2580	2663	-	2574	2131	3010	-	2571
	n	1	-	1	1	1	1	-	5	1	1	-	2
	CV	*	-	*	*	*	*	-	8.2	*	*		24.2
**CP**	Mean	308.3	314.8	284	282	277	302.08	306.34	309.59	463.2	365.58	426.88	446.18
	n	83	42	1	1	1	3	1	132	7	1	2	10
	CV	5.6	7.8	*	*	*	0.4	*	6.5	14.36	-	12,6	14.7
**EE**	Mean	94.58	85.3	145	148	141	89.08	74.35	92.17	52.77	107.99	99.31	69.25
	n	53	38	1	1	1	2	1	97	6	1	2	9
	CV	29	45.5	*	*	*	6.3	*	36	45.9	*	17.5	46.2
**Ash**	Mean	57.37	51.87	44.3	53.2	50.5	49.19	56.81	54.59	25	-	34.14	27.61
	n	48	42	1	1	1	1	1	95	5	-	2	7
	CV	17.7	24.4	*	*	*	*	*	20.9	27.9	-	15.3	27.3
**NDF**	Mean	358.8	391.2	287	343	328	323.78	425.07	368.44	380.11	464.57	467.5	406.04
	n	76	42	1	1	1	3	1	125	7	1	2	10
	CV	15.6	14.9	*	*	*	4.8	*	15.8	27.8	*	18.8	24.7
**ADF**	Mean	124.3	153.4	144	130	122	104.36	166.24	129.54	217.09	169.85	205.48	210.05
	n	76	17	1	1	1	2	1	99	7	1	2	10
	CV	23.4	56.7	*	*	*	33.7	*	34.9	32.2	*	10.7	28.3
**Starch**	Mean	53.38	134.6	82.9	47.1	42.9	39.96	-	81.95	118.02	25.87	-	71.95
	n	59	35	1	1	1	2	-	99	1	1	-	2
	CV	56.2	35.2	*	*	*	36.9	-	65.2	*	*	*	90.5

GE = gross energy; DE = digestible energy; ME = metabolic energy; NE = net energy; CP = crude protein; EE = ether extract; NDF = neutral detergent fiber; ADF = acid detergent fiber. ^1^ Except for GE, whose unit of measurement is kcal/kg, the other analyses are expressed in g/kg of DM. Variables with only one sample are indicated with an asterisk (*). ^2^ Types of distillers’ co-products considered in the current study. DDGS: dried distillers grains with solubles; HP-DDG: high-protein dried distillers grains; ^3^ n = number of samples; ^4^ CV = coefficient of variation, %.

**Table 4:** Chemical compositions of Brazilian corn DDGS and HP-DDGs ^1^.

Composition		Type ^2^	
		DDGS	HP-DDGs
**CP**	Mean	310.8	433.5
	N ^3^	25	1490
	CV ^4^	5.6	5.0
**EE**	Mean	77.2	118.6
	N	23	1480
	CV	16.4	13.2
**Ash**	Mean	43.5	25.9
	N	25	1478
	CV	11.0	25.6
**NDF**	Mean	325.0	306.9
	N	11	1257
	CV	16.4	7.7
**ADF**	Mean	104.4	106.7
	N	11	1257
	CV	30.5	10.5

CP = crude protein; EE = ether extract; NDF = neutral detergent fiber; ADF = acid detergent fiber. ^1^ Chemical analyses (CP, EE, Ash, NDF, and ADF) are expressed in g/kg DM. ^2^ Type of distillers’ co-products: DDGS: dried distillers grains with solubles; HP-DDGs: high-protein dried distillers grains; ^3^ N = number of samples; ^4^ CV = coefficient of variation, %.

**Table 5:** Variability in HP-DDGs among factories and/or manufacturing units ^1^.

Manufacturers ^2^		HP-DDGs			*p*-Value ^3^
		Factory 1.1	Factory 1.2	Factory 2.1	
**CP**	Mean	418.9 ^c^	443.4 ^b^	480.3 ^a^	<0.001
	n ^4^	770	607	113	
	CV ^5^	2.4	2.5	6.3	
**EE**	Mean	127.6 ^a^	114.7 ^b^	75.2 ^c^	<0.001
	N	768	607	105	
	CV	6.4	4.8	20.9	
**Ash**	Mean	27.1 ^b^	22.9 ^c^	33.8 ^a^	<0.001
	N	767	606	105	
	CV	20.6	19.16	36.8	
**NDF**	Mean	303.9 ^b^	309.5 ^b^	317.5 ^a^	<0.001
	N	654	546	57	
	CV	7.7	5.5	17.4	
**ADF**	Mean	104.4 ^b^	110.6 ^a^	94.7 ^c^	<0.001
	N	654	546	57	
	CV	10.7	7.5	19.7	

CP = crude protein; EE = ether extract; NDF = neutral detergent fiber; ADF = acid detergent fiber. ^1^ Chemical analyses (CP, EE, Ash, NDF, and ADF) are expressed in g/kg DM. ^2^ The first number of the manufacturer identifies the factory, and the second number after the dot is the manufacturing unit. ^3^ The *p*-values were calculated using a non-parametric test (Kruskal–Wallis test), followed by multiple comparisons. ^a,b,c^ Within a row, means with different superscripts are affected by manufacturers (*p* < 0.05). ^4^ N = number of samples; ^5^ CV = coefficient of variation, %.

## Data Availability

Data are contained within the article.

## Supplementary Materials

The following supporting information can be downloaded at https://www.mdpi.com/article/10.3390/ani14233455/s1, Table S1: Descriptive analysis of the chemical composition of distillery co-products from the systematic literature review database.
