# Supplementary material for: Variability in Distillers’ Co-Product Compositions and Their Nutritional Availability for Pigs: Insights from a Systematic Literature Review
Source: Animals (Basel). 2024 Nov 29;14(23):3455. doi: 10.3390/ani14233455 (PMC11640343; doi:10.3390/ani14233455)
Supplement: Supplementary file 1 [file animals-14-03455-s001.zip › animals-3234865-supplementary.pdf]

Supplementary material

S1 Table. Descriptive analysis of the chemical composition of distillery co-products from the systematic literature review database.

Variable[1]	Type[2]	Source[3]	n	Mean	CV	Minimum	Maximum
CP	DDG	Corn	6	307.1	13.1	246.2	341.8
	DDGS	Corn	132	309.6	6.5	272.5	368.4
		Mix	12	344.5	7.8	318.0	424.0
		Sorghum	1	281.2		281.2	281.2
		Triticale	1	272.6		272.6	272.6
		Wheat	7	367.1	12.4	320.0	445.0
	FWS	Corn	3	180.0	12.8	158.4	204.3
	HP-DDG	Corn	10	446.2	14.7	365.6	600.0
	HP-DDGS	Corn	4	477.8	12.6	423.3	547.8
		Sorghum	1	522.2		522.2	522.2
	HYP	Corn	2	497.9	14.0	448.6	547.3
EE	DDG	Corn	1	95.1		95.1	95.1
	DDGS	Corn	97	92.2	36.0	12.4	148.0
		Mix	9	81.2	28.6	36.4	98.6
		Sorghum	1	88.9		88.9	88.9
		Triticale	1	56.4		56.4	56.4
		Wheat	4	64.4	37.3	29.0	82.2
	FWS	Corn	3	101.6	29.0	71.6	130.5
	HP-DDG	Corn	9	69.2	46.2	19.7	111.6
	HP-DDGS	Corn	4	61.0	76.1	11.9	100.9
		Sorghum	1	34.0		34.0	34.0
	HYP	Corn	2	99.8	70.1	50.4	149.3
Ash	DDG	Corn	4	34.5	60.2	16.3	63.7
	DDGS	Corn	95	54.6	20.9	16.3	90.7
		Mix	10	57.4	13.0	46.2	71.9
		Sorghum	-				
		Triticale	-				
		Wheat	6	49.7	13.3	37.5	56.6
	FWS	Corn	2	52.7	5.5	50.7	54.8
	HP-DDG	Corn	7	27.6	27.3	15.7	37.8
	HP-DDGS	Corn	3	43.3	69.6	25.6	78.0
		Sorghum	1	54.3		54.3	54.3
	HYP	Corn	2	55.3	11.2	50.9	59.6
NDF	DDG	Corn	6	494.6	17.0	393.5	610.9
	DDGS	Corn	125	368.4	15.8	139.6	593.3
		Mix	12	323.0	9.6	275.0	374.0
		Sorghum	1	387.4		387.4	387.4
		Triticale	1	357.2		357.2	357.2
		Wheat	7	321.2	16.5	240.0	388.5
	FWS	Corn	3	385.8	13.5	352.8	446.1

	HP-DDG	Corn	10	406.0	24.7	172.8	529.8
	HP-DDGS	Corn	3	316.2	1.5	311.1	319.9
		Sorghum	1	221.0		221.0	221.0
	HYP	Corn	2	184.3	62.1	103.4	265.2
ADF	DDG	Corn	6	266.2	35.2	191.2	423.1
	DDGS	Corn	99	129.5	34.9	19.2	430.0
		Mix	10	172.3	24.3	123.0	242.9
		Sorghum	1	201.9		201.9	201.9
		Triticale	1	153.3		153.3	153.3
		Wheat	6	198.9	37.7	72.9	273.7
	FWS	Corn	3	108.8	35.0	80.0	152.0
	HP-DDG	Corn	10	210.0	28.3	91.7	275.6
	HP-DDGS	Corn	3	167.7	7.5	158.9	182.2
		Sorghum	1	189.6		189.6	189.6
	HYP	Corn	2	89.0	58.0	52.5	125.5

[1] Variables: CP = crude protein; EE = ether extract, NDF = neutral detergent fiber, ADF = acid detergent fiber. The unit of measurement of the variables is g/kg.

[2] Types of distillery co-products considered in the current paper. DDGS: dry distillated grain with soluble; DDG: dry distillated grain; HP-DDGS: high protein dry distillated grain with soluble; HP-DDG: high protein: dry distillated grain; FWS: fiber with soluble; HYP: high yeast and protein.

[3] Source: raw materials used to produce the distillery co-products.

n = number of samples; CV = coefficient variation.

S2 Table. Energy content from the systematic literature review database according to the types of distillery co-products and grain sources used in fermentation.

Energy[1]	Type[2]	Source[3]	n	Mean	CV	Minimum	Maximum
GE	DDG	Corn	4	5,384	8.0	4,988	5,857
	DDGS	Corn	107	5,168	4.4	4,445	5,873
		Mix	10	4,971	2.4	4,705	5,087
		Sorghum	1	5,295			
		Triticale	1	5,298			
		Wheat	5	4,976	1.3	4,872	5,040
	FWS	Corn	3	4,925	5.4	4,629	5,152
	HP-DDG	Corn	8	5,488	3.0	5,293	5,738
	HP-DDGS	Corn	3	5,407	0.3	5,392	5,425
		Sorghum	1	5,108			
	HYP	Corn	2	5,414	1.7	5,348	5,479
DE	DDGS	Corn	77	3,663	6.4	3,045	4,292
		Mix	1	4,038			
		Sorghum	1	3,520			
		Triticale	1	3,720			
		Wheat	1	4,019			
	FWS	Corn	2	3,146	3.7	3,063	3,229
	HP-DDG	Corn	8	3,946	22.3	2,267	5,043
	HP-DDGS	Corn	3	4,665	5.2	4,494	4,945
		Sorghum	1	3,878			
	HYP	Corn	2	4,246	2.1	4,182	4,309
ME	DDGS	Corn	63	3,468	8.0	2,850	3,899
		Sorghum	1	3,228			
		Triticale	1	3,315			
	FWS	Corn	1	3,143			
	HP-DDG	Corn	6	3,654	25.0	2,166	4,690
	HP-DDGS	Corn	1	4,669			
		Sorghum	1	3,549			
	HYP	Corn	2	4,045	2.5	3,972	4,118
NE	DDGS	Corn	5	2,574	8.2	2,208	2,747
		Mix	7	2,272	4.1	2,114	2,384
		Wheat	3	2,183	9.2	1,980	2,381
	HP-DDG	Corn	2	2,571	24.2	2,131	3,010
		Sorghum	1	2,256			

[1] Variables: GE = gross energy; DE = digestible energy; ME = metabolic energy; EL = energy liquid. The unit of measurement of the variables is kcal/kg of dry matter

[2] Types of distillery co-products considered in the current paper. DDGS: dry distillated grain with soluble; DDG: dry distillated grain; HP-DDGS: high protein dry distillated grain with soluble; HP-DDG: high protein: dry distillated grain; FWS: fiber with soluble; HYP: high yeast and protein.

[3] Source: raw materials used to produce the distillery co-products.

n = number of samples; CV = coefficient variation.

Supplementary references from Table 1

1. Acosta, J.P.; Espinosa, C.D.; Jaworski, N.W.; Stein, H.H. Corn protein has greater concentrations of digestible amino acids and energy than low-oil corn distillers dried grains with solubles when fed to pigs but does not affect the growth performance of weanling pigs. *J. Anim. Sci.* **2021**, *99*: skab175. https://doi.org/10.1093/jas/skab175
2. Adebiyi, A.O.; Ragland, D.; Adeola, O.; Olukosi, O.A.Apparent or standardized ileal digestibility of amino acids of diets containing different protein feedstuffs fed at two crude protein levels for growing pigs. Asian-Australas. *J. Anim. Sci.* **2015**, *28,* 1327-1334. https://doi.org/10.5713/ajas.14.0914
3. Adeola, O.; Kong, C. Energy value of distillers dried grains with solubles and oilseed meals for pigs. *J. Anim. Sci.* **2014**, *92,* 164-170. https://doi.org/10.2527/jas.2013-6662
4. Adeola, O.; Ragland, D. Ileal digestibility of amino acids in co-products of corn processing into ethanol for pigs. *J. Anim. Sci.* **2012**, *90*, 86-88. https://doi.org/ 10.2527/jas.51661
5. Adeola, O.; Ragland, D. Comparative ileal amino acid digestibility of distillers' grains for growing pigs. *Anim. Nutr.* **2016**, *2*, 262-266. https://doi.org/ 10.1016/j.aninu.2016.07.008
6. Agyekum, A.K.; Regassa, A.; Kiarie, E.; Nyachoti, C.M. Nutrient digestibility, digesta volatile fatty acids, and intestinal bacterial profile in growing pigs fed a distillers dried grains with solubles containing diet supplemented with a multi-enzyme cocktail. *Anim. Feed. Sci. Technol*. **2016,** *212,* 70-80. https://doi.org/10.1016/j.anifeedsci.2015.12.006
7. Almeida, F.N.; Htoo, J.K.; Thomson, J.; Stein, H.H. Amino acid digestibility of heat damaged distillers dried grains with solubles fed to pigs. *J. Anim. Sci. Biotechnol*. **2013**, *4*, 1-10. https://doi.org/10.1186/2049-1891-4-44
8. Almeida, F.N.; Petersen, G.I.; Stein, H.H. Digestibility of amino acids in corn, corn co-products, and bakery meal fed to growing pigs. *J. Anim. Sci.* **2011**, *89*, 4109-4115. https://doi.org/10.2527/jas.2011-4143
9. Almeida, F.N.; Stein, H.H. Effects of graded levels of microbial phytase on the standardized total tract digestibility of phosphorus in corn and corn co-products fed to pigs. *J. Anim. Sci.* **2012,** *90*, 1262-1269. https://doi.org/10.2527/jas.2011-4144
10. Baker, S.R.; Kim, B.G.; Stein, H.H. Comparison of values for standardized total tract digestibility and relative bioavailability of phosphorus in dicalcium phosphate and distillers dried grains with solubles fed to growing pigs. *J. Anim. Sci.* **2013**, *91*, 203-210. https://doi.org/ 10.2527/jas.2010-3776
11. Boucher, M.; Zhu, C.; Holt, S.; Huber, L.A. Physiochemical characterization and energy contents of novel corn ethanol co-product streams, with and without inclusion of a multi-carbohydrase enzyme blend, for growing pigs. *Can. J. Anim. Sci.* **2021**, *101*, 353-361. https://doi.org/10.1139/cjas-2020-0144
12. Cristobal, M.; Acosta, J.P.; Lee, S.A.; Stein, H.H. A new source of high-protein distillers dried grains with solubles (DDGS) has greater digestibility of amino acids and energy, but less digestibility of phosphorus, than de-oiled DDGS when fed to growing pigs. *J. Anim. Sci.* **2020**, *98*, skaa200. https://doi.org/10.1093/jas/skaa200
13. Curry, S.M.; Blavi, L.; Wiseman, J.; Stein, H.H. Effects of distillers dried grains with solubles on amino acid digestibility, growth performance, and carcass characteristics of growing pigs. *Transl. Anim. Sci.* **2019**, *3*, 641-653. https://doi.org/ 10.1093/tas/txz005
14. Curry, S.M.; Navarro, D.M.D.L.; Almeida, F.N.; Almeida, J.A.S.; Stein, H.H. Amino acid digestibility in low-fat distillers dried grains with solubles fed to growing pigs. J. *Anim. Sci. Biotechnol.* **2014**, *5*, 1-7. https://doi.org/10.1186/2049-1891-5-27
15. Espinosa, C.D.; Lee, S.A.; Stein, H.H. Digestibility of amino acids, energy, acid hydrolyzed ether extract, and neutral detergent fiber, and concentration of digestible and metabolizable energy in low-oil distillers dried grains with solubles fed to growing pigs. *Transl. Anim. Sci.* **2019**, *3*, 662-675.https://doi.org/10.1093/tas/txz025
16. Fastinger, N.D.; Mahan, D.C. Determination of the ileal amino acid and energy digestibilities of corn distillers dried grains with solubles using grower-finisher pigs. *J. Anim. Sci.* **2006**, *84*, 1722-1728. https://doi.org/10.2527/jas.2005-308
17. Graham, A.B.; Goodband, R.D.; Tokach, M.D.; Dritz, S.S.; DeRouchey, J.M.; Nitikanchana, S. The effects of medium-oil dried distillers grains with solubles on growth performance, carcass traits, and nutrient digestibility in growing-finishing pigs. J. Anim. Sci. **2014**, 92, 604-611. https://doi.org/10.2527/jas.2013-6798
18. Guo, L.; Piao, X.; Li, D.; Li, S. The apparent digestibility of corn by-products for growing-finishing pigs in vivo and in vitro. *Asian-australas J. Anim. Sci.* **2004**, *17*, 379-385. https://doi.org/10.5713/AJAS.2004.379

19. Huang, Z.; Urriola, P.E.; Shurson, G.C. Prediction of digestible and metabolizable energy of corn distillers dried grains with solubles for growing pigs using in vitro digestible nutrients. *J. Anim. Sci.* **2018**, *96*, 1818-1824.https://doi.org/ 10.1093/jas/sky102
20. Jacela, J.Y.; Frobose, H.L.; DeRouchey, J.M.; Tokach, M.D.; Dritz, S.S.; Goodband, R.D.; Nelssen, J.L. Amino acid digestibility and energy concentration of high-protein corn dried distillers grains and high-protein sorghum dried distillers grains with solubles for swine. *J. Anim. Sci.* **2010,** *88*, 3617
21. Kerr, B.J.; Dozier Iii, W.A.; Shurson, G.C. Effects of reduced-oil corn distillers dried grains with solubles composition on digestible and metabolizable energy value and prediction in growing pigs. *J. Anim. Sci.* **2013**, *91*, 3231-3243. https://doi.org/10.2527/jas.2013-6252
22. Kim, B.G.; Liu, Y.; Stein, H.H. Effects of ileal digesta collection time on standardized ileal digestibility of amino acids in corn, soybean meal, and distillers dried grains with solubles fed to growing pigs. *J. Anim. Sci.* **2017**, *95*, 789-798. https://doi.org/ 10.2527/jas.2016.1082
23. Kim, B.G.; Petersen, G.I.; Hinson, R.B.; Allee, G.L.; Stein, H.H. Amino acid digestibility and energy concentration in a novel source of high-protein distillers dried grains and their effects on growth performance of pigs. *J. Anim. Sci.* **2009**, *87*, 4013-4021. https://doi.org/10.2527/jas.2009-2060
24. Li, P.; Xu, X.; Zhang, Q.; Liu, J.D.; Li, Q.Y.; Zhang, S.; Ma, X.K.; Piao, X.S. Effect of different inclusion level of condensed distillers solubles ratios and oil content on amino acid digestibility of corn distillers dried grains with solubles in growing pigs. *Asian-Australas. J. Anim. Sci.* **2015a**, *28*, 102-110. https://doi.org/ 10.5713/ajas.14.0161
25. Li, P.; Li, D.F.; Zhang, H.Y.; Li, Z.C.; Zhao, P.F.; Zeng, Z.K.; Xu, X.; Piao. X.S. Determination and prediction of energy values in corn distillers dried grains with solubles sources with varying oil content for growing pigs. *J. Anim. Sci*. **2015b**, *93,* 3458-3470. https://doi.org/ 10.2527/jas.2014-8782
26. Liang, G.; Li, D.; Wang, F.; Dai, J.; Yang, W. Evaluation of apparent ileal digestibility of amino acids in Chinese corn by-products for growing-finishing pigs. *Arch. Anim. Nutr.* **2003**, *57*, 117-125. https://doi.org/10.1080/0003942031000107316
27. Liu, P.; Souza, L.W.O.; Baidoo, S.K.; Shurson, G.C. Impact of distillers dried grains with solubles particle size on nutrient digestibility, DE and ME content, and flowability in diets for growing pigs. *J. Anim. Sci.* **2012**, *90*, 4925-4932. https://doi.org/ 10.2527/jas.2011-4604
28. Navarro, D.M.D.L.; Bruininx, E.M.A.M.; de Jong, L.; Stein, H.H. Effects of physicochemical characteristics of feed ingredients on the apparent total tract digestibility of energy, DM, and nutrients by growing pigs. *J. Anim. Sci.* **2018**, *96*, 2265-2277.https://doi.org/ 10.1093/jas/sky149
29. Pahm, A.A.; Pedersen, C.; Hoehler, D.; Stein, H.H. Factors affecting the variability in ileal amino acid digestibility in corn distillers dried grains with solubles fed to growing pigs. *J. Anim. Sci.* **2008**, *86*, 2180-2189. https://doi.org/10.2527/jas.2008-0868
30. Pahm, A.A.; Pedersen, C.; Stein, H.H. Standardized ileal digestibility of reactive lysine in distillers dried grains with solubles fed to growing pigs. *J. Agric. Food. Chem*. **2009**, *57*, 535-539. https://doi.org/10.1021/jf802047d
31. Park, C.S.; Ragland, D.; Adeola, O. Amino acid digestibility of corn distillers' dried grains with solubles with the addition of casein in pigs. *J. Anim. Sci.* **2018**, *96*, 4674-4684. https://doi.org/ 10.1016/j.animal.2020.100147
32. Park, C.S.; Ragland, D.; Adeola, O. Amino acid digestibility in corn distillers' dried grains with solubles in pigs at different dietary levels of casein and test ingredient. *Animal.* **2021,** *15*, 100147. https://doi.org/10.1016/j.animal.2020.100147
33. Paula, V.R.C.; Milani, N.C.; Azevedo, C.P.F.; Sedano, A.A.; Souza, L.J.; Mike, B.P.; Shurson, G.C.; Ruiz, U.S. Comparison of digestible and metabolizable energy and digestible phosphorus and amino acid content of corn ethanol co-products from Brazil and the United States produced using fiber separation technology for swine. *J. Anim. Sci*. **2021**, *99*, skab126. https://doi.org/10.1093/jas/skab126.
34. Petersen, G.I.; Liu, Y.; Stein, H.H. Coefficient of standardized ileal digestibility of amino acids in corn, soybean meal, corn gluten meal, high-protein distillers dried grains, and field peas fed to weanling pigs. *Anim. Feed. Sci. Technol.* **2014**, *188*, 145-149. https://doi.org/10.1016/j.anifeedsci.2013.11.002
35. Ren, P.; Zhu, Z.; Dong, B.; Zang, J.; Gong, L. Determination of energy and amino acid digestibility in growing pigs fed corn distillers' dried grains with solubles containing different lipid levels. *Arch. Anim. Nutr.* **2011,** *65*, 303-319. https://doi.org/10.1080/1745039x.2011.588849
36. Rho, Y.; Zhu, C.; Kiarie, E.; de Lange, C.F.M. Standardized ileal digestible amino acids and digestible energy contents in high-protein distillers dried grains with solubles fed to growing pigs. *J. Anim. Sci.* **2017**, *95*, 3591-3597. https://doi.org/ 10.2527/jas.2017.1553
37. Rodriguez, D.A.; Lee, S.A.; Stein, H.H. Digestibility of amino acids, but not fiber, fat, or energy, is greater in cold-fermented, low-oil distillers dried grains with solubles (DDGS) compared with conventional DDGS fed to growing pigs. *J Anim Sci*. **2020,** *98,* skaa297.https://doi/org/10.1093/jas/skaa297

38. Soares, J.A.; Stein, H.H.; Singh, V.; Shurson, G.S.; Pettigrew, J.E. Amino acid digestibility of corn distillers dried grains with solubles, liquid condensed solubles, pulse dried thin stillage, and syrup balls fed to growing pigs. *J. Anim. Sci*. **2012**, *90*, 1255-1261. https://doi.org/ 10.2527/jas.2010-3691
39. Stein, H.H.; Gibson, M.L.; Pedersen, C.; Boersma, M.G. Amino acid and energy digestibility in ten samples of distillers dried grain with solubles fed to growing pigs. *J. Anim. Sci.* **2006**, *84*, 853-860. https://doi.org/10.2527/2006.844853x
40. Tanghe, S.; De Boever, J.; Ampe, B.; De Brabander, D.; De Campeneere, S.; Millet, S. Nutrient composition, digestibility and energy value of distillers dried grains with solubles and condensed distillers solubles fed to growing pigs and evaluation of prediction methods. *Anim. Feed. Sci. Technol.* **2015,** *210*, 263-275. https://doi.org/10.1016/j.anifeedsci.2015.10.015
41. Urriola, P.E.; Stein, H.H.. Effects of distillers dried grains with solubles on amino acid, energy, and fiber digestibility and on hindgut fermentation of dietary fiber in a corn-soybean meal diet fed to growing pigs. *J. Anim. Sci.* **2010,** *88*, 1454-1462. https://doi.org/10.2527/jas.2009-2162
42. Widmer, M.R.; McGinnis, L.M.; Stein, H.H. Energy, phosphorus, and amino acid digestibility of high-protein distillers dried grains and corn germ fed to growing pigs. *J. Anim. Sci*. **2007**, *85*, 2994-3003. https://doi.org/10.2527/jas.2006-840
43. Widyaratne, G.R.; Zijlstra, R.T. Nutritional value of wheat and corn distillers dried grain with solubles: Digestibility and digestible contents of energy, amino acids and phosphorus, nutrient excretion and growth performance of grower-finisher pigs. *Can. J. Anim. Sci*. **2007,** *87*, 103-114. https://doi.org/10.4141/A05-070
44. Xie, F.; Li, Y.K.; Zhao, J.B.; Li, Z.C.; Liu, L.; Cao, Y.H.; Zhang, S. Comparative digestibility of energy and nutrients in four fibrous ingredients fed to barrows at three different initial body weights. *Can J Anim Sci.* **2019**; *99*: 315-325. https://doi.org/10.1139/cjas-2017-0094
45. Yang, Y.; Kiarie, E.; Slominski, B.A.; Brule-Babel, A.; Nyachoti, C.M. Amino acid and fiber digestibility, intestinal bacterial profile, and enzyme activity in growing pigs fed dried distillers grains with solubles-based diets. *J. Anim. Sci.* **2010,** *88*, 3304-3312. https://doi.org/10.2527/jas.2009-2318
46. Yang, Z.; Palowski, A.; Jang, J.C.; Urriola, P.E.; Shurson, G.C. Determination, comparison, and prediction of digestible energy, metabolizable energy, and standardized ileal digestibility of amino acids in novel maize co-products and conventional distillers dried grains with solubles for swine. *Anim. Feed. Sci. Technol.* **2021**, *282*, 115149. https://doi.org/10.1016/j.anifeedsci.2021.115149
47. Zangaro, C.A.; Woyengo, T.A. Nutrient digestibility of heat- or heat plus citric acid-pretreated dried distillers grains with solubles for pigs. *Anim. Feed. Sci. Technol.* **2022**, *285*, 115238. https://doi.org/10.1016/j.anifeedsci.2022.115238
48. Zhang, Z.; Zhang, G.; Zhang, S.; Zhao, J. Effects of extrusion on energy contents and amino acid digestibility of corn DDGS and full-fat rice bran in growing pigs. *Animals.* **2022**, *12,* 579. https://doi.org/10.3390/ani12050579
